# Triptolide Induces Glioma Cell Autophagy and Apoptosis via Upregulating the ROS/JNK and Downregulating the Akt/mTOR Signaling Pathways

**DOI:** 10.3389/fonc.2019.00387

**Published:** 2019-05-14

**Authors:** Xihong Liu, Peiyuan Zhao, Xiujuan Wang, Lei Wang, Yingjun Zhu, Wei Gao

**Affiliations:** ^1^School of Traditional Chinese Medicine, Capital Medical University, Beijing, China; ^2^Basic Discipline of Integrated Chinese and Western Medicine, Henan University of Chinese Medicine, Henan, China; ^3^School of Pharmaceutical Sciences, Capital Medical University, Beijing, China; ^4^Advanced Innovation Center for Human Brain Protection, Capital Medical University, Beijing, China

**Keywords:** triptolide, apoptosis, autophagy, LC3, ROS

## Abstract

Apoptosis and autophagy are the two prominent forms of developmental cell death, and researches have shown that crosstalk exists between these two processes. A prior study demonstrated that triptolide inhibited the proliferation of malignant glioma cells. However, whether apoptosis and autophagy participate in the inhibitory effect of triptolide in glioma cells has not been clarified. In the present study, we demonstrated that triptolide potently inhibited the growth of glioma cells by inducing cell cycle arrest at the G2/M phase. Additionally, the treatment with triptolide induced apoptosis and autophagy in various glioma cell lines. Triptolide-induced autophagy may have tumor-supporting effects. Autophagy and apoptosis could cross-inhibit each other in glioma cells treated with triptolide. Moreover, we found that triptolide induced ROS production and JNK activation and inhibited the activity of Akt and mTOR. Finally, we demonstrated that triptolide suppressed tumor growth in an orthotopic xenograft glioma model. Collectively, these data indicated that triptolide induced G2/M phase arrest, apoptosis, and autophagy via activating the ROS/JNK and blocking the Akt/mTOR signaling pathways in glioma cells. Triptolide may be a potential anti-tumor drug targeting gliomas.

## Introduction

Gliomas are common and lethal malignant primary brain tumors with a poor prognosis that exhibit strong invasion, rapid progression, and vulnerability to relapse (1–3). The treatment of gliomas has become multi-modality over the past few decades. The current standard of care for gliomas is surgery, followed by radiotherapy and the first-line chemotherapy agent temozolomide (TMZ). Unfortunately, this treatment intervention has little impact on the survival rate of patients, during which the survival rate has only increased by 3–6 months (4). Therefore, it is urgent to explore alternative treatments to provide new hope for the treatment of gliomas.

Triptolide, which was the first diterpenoid triepoxide lactone isolated from *Tripterygium wilfordii* Hook F, has been recognized as a principal component responsible for the biological activities of the plant (5). Triptolide has been demonstrated to possess a wide range of biological activities, such as anticancer, immunosuppressive, contraceptive, anti-angiogenic, and anti-inflammatory activities (6–10). In 2007, in addition to celastrol, artemisinin, capsaicin, and curcumin, triptolide was deemed to be a “poster child” due to its power and potential of transforming traditional medicine into modern medicine (11). Mounting evidence suggests that triptolide possesses potent broad-spectrum anticancer activities. Triptolide kills almost all cancer cells originating from the prostate, colon, breast, blood, lung and kidney, and some derivatives of triptolide are presently under clinical evaluation (12–15). Previous research has demonstrated that triptolide inhibits the proliferation of glioma cells *in vitro* and *in vivo*; however, the underlying molecular mechanisms remain unclear (16).

Apoptosis and autophagy are two main forms of programmed cell death (PCD), they may jointly decide the fate of cells of malignant neoplasms. Apoptosis, or type I PCD, is critical for the development and homeostasis of multicellular organisms and is characterized by specific morphological and biochemical changes of dying cells, including cell shrinkage, nuclear condensation and fragmentation (17). Autophagy or type II PCD is an evolutionarily conserved catabolic process in which cellular material is delivered by double-membrane structures called autophagosomes to lysosomes for degradation (18). In general, apoptosis invariably contributes to cell death, whereas autophagy can play either pro-survival or pro-death roles during different stages of tumor development. For example, on the one hand, autophagy improves the adaptability of cancer cells to resist apoptosis under pressure. On the other hand, autophagy reduces metastasis by limiting tumor necrosis and preventing inflammatory immune cell infiltration. In addition, excessive autophagy induces the death of cancer cells. Of note, apoptosis and autophagy are not mutually exclusive pathways, there still exist intricate interrelationships between them. They have proved to be synergistic or antagonistic (19). Therefore, it is crucial to further elucidate the function of triptolide-induced autophagy and the relationship between apoptosis and autophagy in glioma cells.

Reactive oxygen species (ROS), which are active forms of oxygen, have been described as toxic molecules with high biological activity and are involved in the biological effects of many agents (20). At low levels, ROS exhibit beneficial effects, whereas their excessive accumulation can promote cancer (21). The mitochondria represent a major source of oxidative stress, and the JNK signaling pathway is one of the numerous downstream cascades of the ROS signaling pathway and is closely associated with cell proliferation, differentiation, mitosis, survival, and apoptosis (22). Furthermore, the Akt/mTOR signaling pathway has emerged as one of the major regulatory pathways of cell survival, growth, proliferation, and protein synthesis in both normal cells and cancer cells (23, 24). Previous studies have suggested that the Akt/mTOR cascade is involved in the processes of autophagy and apoptosis (25).

In the present study, we investigated the antitumor effects and possible mechanisms underlying the impact of triptolide on glioma cells both *in vitro* and *in vivo*. We elucidated that triptolide induced G2/M-phase arrest, apoptosis, and autophagy in glioma cells by activating the ROS/JNK and inhibiting Akt/mTOR signaling pathways. In addition, we found that triptolide-induced autophagy played a role in promoting cell survival. Triptolide-induced apoptosis and autophagy may inhibit each other.

## Materials and Methods

### Cell Lines and Reagents

U251 (Human, CRC/PUMC, 3111C0001CCC000058), U87-MG (Human, CRC/PUMC, 3111C0001CCC000208), and C6 (Rat, CRC/PUMC, 3111C0001CCC000131) cells were purchased from the Cell Resource Center, IBMS, CAMS/PUMC. The cells were cultured in minimum Eagle's medium (MEM; Corning, NY, USA), MEM-NEAA or F10 (Gibco, UK) supplemented with 10% (v/v) fetal bovine serum (Gibco, UK), 100 U/ml penicillin and 100 μg/ml streptomycin at 37°C in a humidified incubator with 5% CO_2_. Triptolide (purity >99%) was purchased from Pharmacodia Co., Ltd (Beijing, China). The stock solutions (100 μM) were prepared with DMSO and stored in the dark at −20°C. TMZ were obtained from Sigma (Sigma, St. Louis, MO, USA). All other chemicals were purchased from Selleckchem (Houston, TX, USA). The primary antibodies against cleaved caspase-3 (cle-cap.3), p62, LC3A/B (LC3), CyclinB1, and phospho-Cdc2 (p-Cdc2) were obtained from Cell Signaling Technology (Beverly, MA, USA). The primary antibodies against cleaved caspase-8 (cle-cap.8), cleaved caspase-9 (cle-cap.9), cleaved-PARP (cle-PARP), Beclin-1, Akt, phospho-Akt (p-Akt), JNK, phospho-JNK (p-JNK), mTOR, phospho-mTOR (p-mTOR), Chk2, phospho-Chk2 (p-Chk2), Cdc25C, phospho-Cdc25C (p-Cdc25c), and p21 were purchased from Abcam (Cambridge, MA, UK). The primary antibody against β-actin was purchased from Proteintech (Chicago, IL, USA). The secondary antibodies were obtained from Cell Signaling Technology. The plasmid ptfLC3 (mRFP-EGFP-LC3B) was purchased from the Addgene Repository (http://www.addgene.org).

### Cell Viability Assay

The cells were seeded in 96-well plates at a density of 2,500–5,000 cells per well and treated with various concentrations of triptolide (30, 100, 300, or 1,000 nM) for 12 to 48 h. Then, the cell viability was assessed with a Cell Counting Kit-8 (CCK8; Dojindo, Kumamoto, Japan) according to the provided protocol. Briefly, the culture medium was discarded, and each well was supplemented with a mixture of CCK8 reagent and MEM and incubated for 0.5–1 h. The optical density (OD) value at 450 nm was determined by a microplate reader (Molecular Devices, Sunnyvale, CA, USA). The cell viability was normalized to that of the controls.

### Colony Formation Assay

U251, U87-MG, and C6 cells were seeded in 6-well plates at a density of 100–400 cells/well and treated with gradient concentrations of triptolide (3, 10, 30, or 100 nM) for the indicated times. After ~10 days, the medium was discarded, and the cells were washed 3 times with PBS once they formed visible colonies. Then, the colonies were stained with 0.1% crystal violet for 15 min after being fixed with 4% paraformaldehyde. The colonies were defined as clusters of ≥ 50 cells, and the images were captured with a camera. The colonies were counted and normalized to the number of colonies in the control group.

### Cell Cycle Distribution Analysis by Flow Cytometry

The role of triptolide in the DNA content and cell cycle progression was analyzed with a propidium iodide (PI)/RNase staining buffer (BD Biosciences, San Diego, CA, USA). Briefly, after treatment with triptolide or DMSO for 24 h, the cells were harvested, washed 3 times with ice-cold PBS and fixed with 75% ethanol at −20°C overnight. Then, the cells were resuspended in PBS and stained with PI/RNase staining buffer for 15 min. The cell cycle phases were monitored by flow cytometry (Novocyte, ACEA Biosciences, San Diego, CA, USA), and the cycle distribution was analyzed with NovoExpress software.

### Cell Apoptosis Assay

The characteristic morphological changes associated with the treatment-induced apoptosis in the glioma cells were observed by fluorescence microscopy using Hoechst 33342 staining. The cells were treated, fixed with 4% paraformaldehyde for 15 min and stained with Hoechst 33342 solution for 30 min at room temperature in the dark. The apoptotic morphology of the glioma cells was observed under a fluorescence microscope (Olympus, Tokyo, Japan). Apoptosis was determined using a FITC Annexin V Apoptosis Detection Kit I (BD Biosciences) according to the provided protocol. In brief, 2.5 × 10^5^ cells were subcultured in 6-well plates and treated with triptolide for an additional 24 h. Then, the cells were harvested, washed twice with cold PBS, and resuspended in 1 × binding buffer. Aliquots of 10^5^ cells were stained with 5 μl of FITC Annexin V and 5 μl of PI. The samples were analyzed using a flow cytometer (LSRFortessa SORP, BD Biosciences, San Jose, CA, USA). The mitochondrial membrane potential (MMP) of the cells was measured using a Mitochondrial Membrane Potential Assay Kit with JC-1 (Beyotime, Jiangsu, China) according to the manufacturer's instructions. The cells cultured in 6-well plates were harvested and resuspended in 500 μl of MEM, and then, 500 μl of JC-1 staining solution were added for 20 min at 37°C in a CO_2_ incubator. The stained cells were washed twice with PBS and analyzed by flow cytometry, and the ratio of green to red fluorescence intensity was determined.

### Measurement of Intracellular ROS

The intracellular ROS generation was determined by using an ROS Assay Kit (Beyotime). Tumor cells treated with the indicated compounds for 12 h were stained with 10 μM DCFH-DA at 37°C for 30 min. The ROS levels were determined under a fluorescence microscope (Leica, Wetzlar, Germany) using a flow cytometer (BD Biosciences).

### Cell Transfection and mRFP-EGFP-LC3B Assay

U251 cells were transiently transfected with 0.8 μg of mRFP-EGFP-LC3B plasmid using Lipofectamine 2,000 (Invitrogen, Carlsbad, CA, USA) according to the manufacturer's instructions. After the transfection, the cultured cells were treated with different chemicals for 24 h and then incubated with DAPI for 15 min. After the treatment, the cells were fixed with 4% paraformaldehyde in PBS. The images were captured under a confocal laser scanning microscope (Leica).

### Western Blotting

The cells and tissue samples were lysed in ice-cold RIPA buffer. The lysates were normalized using an Enhanced BCA Protein Assay Kit (Beyotime) according to the manufacturer's instructions. The proteins were separated by 10–15% SDS-PAGE and transferred to a PVDF membrane. The membrane was blocked in 5% non-fat milk and probed with the indicated primary antibodies at 4°C overnight. The proteins were detected by incubation with species-specific peroxidase-conjugated secondary antibodies. The immunoreactive bands were detected with a Chemiluminescence Kit (Millipore, Plano, TX, USA) and visualized using a Vilber Fusion FX6-XT imaging system.

### *In vivo* Evaluation of Antitumor Activity

All animal experiments were performed according to the guidelines of the Animal Experiments and Experimental Animal Welfare Committee of Capital Medical University (Approval number: AEEI-2017-119). Healthy male athymic nude mice (BALB/c, nu/nu, 6–8 weeks old, 18–20 g) were purchased from Beijing Vital River Laboratory Animal Technology Co., Ltd. All mice were kept under specific pathogen-free conditions and housed in a room under controlled temperature (22 ± 3°C), humidity (40–50%), and light (12 h light/dark cycle) conditions. Sterilized commercial standard solid rodent chow and water were provided *ad libitum*. Aliquots of 3.5 × 10^5^ U251 cells in a volume of 5 μl were injected stereotactically into the right striatum (2 mm lateral and 1 mm anterior to bregma, 3 mm deep) using a small animal stereotactic frame (RWD Life Science, Shenzhen, People's Republic of China). Seven days after the injection, T2-weighted MRI images of the mouse brains were obtained. The mice were randomly divided into the vehicle control, 0.1 mg/kg triptolide, 0.2 mg/kg triptolide, 0.4 mg/kg triptolide, and TMZ (20 mg/kg) groups (8 mice/group), and the corresponding medicine was intraperitoneally administered every other day for a total of seven treatments. The mice were weighed, and their basic condition was observed. At 7 and 14 days after treatment, the tumor volumes were calculated based on the T2-weighted MRI images with the formula V = A × B, where A represents the total tumor area in each tumor layer, and B indicates the thickness of each slice. At 14 days, all mice were sacrificed, and their brains and important organs were collected for further study.

### TUNEL Apoptosis Assay

To detect and quantify the apoptotic response of the tumor tissues, a TUNEL detection kit (Roche Diagnostics, Mannheim, Germany) was used according to the manufacturer's instructions. In brief, paraffin-embedded brain tissues from treated nude mice were deparaffinized and dehydrated. After fixing the tissues, permeabilizing the cell membranes and equilibrating the cells, the slides were incubated with the TUNEL reaction mixture for 1 h at 37°C in a humidified chamber. After washing, the slides were visualized using fluorescence microscopy.

### Histopathology and Immunohistochemistry

Sections (4-μm thick) of embedded primary tumor, heart, liver, spleen, lung, and kidney were stained for a routine histological examination and morphometric analysis. The brain slices were immunostained with the Ki67 (1:400), phospho-JNK (1:100), and cleaved caspase-3 (1:100) antibodies. The digital images were captured under a standard light microscope (Leica).

### Statistical Analysis

All data in this study are presented as the means ± SD from at least 3 independent experiments. All data were analyzed using SPSS version 21.0 software (IBM Corporation, Chicago, USA). One-way ANOVA, Fisher's exact test and Mann-Whitney U-test were used. A *P* < 0.05 indicated statistical significance.

## Results

### Triptolide Is Cytotoxic to Glioma Cells via the Induction of Cell Death and G2/M Cell Cycle Arrest

**Figure 1:**
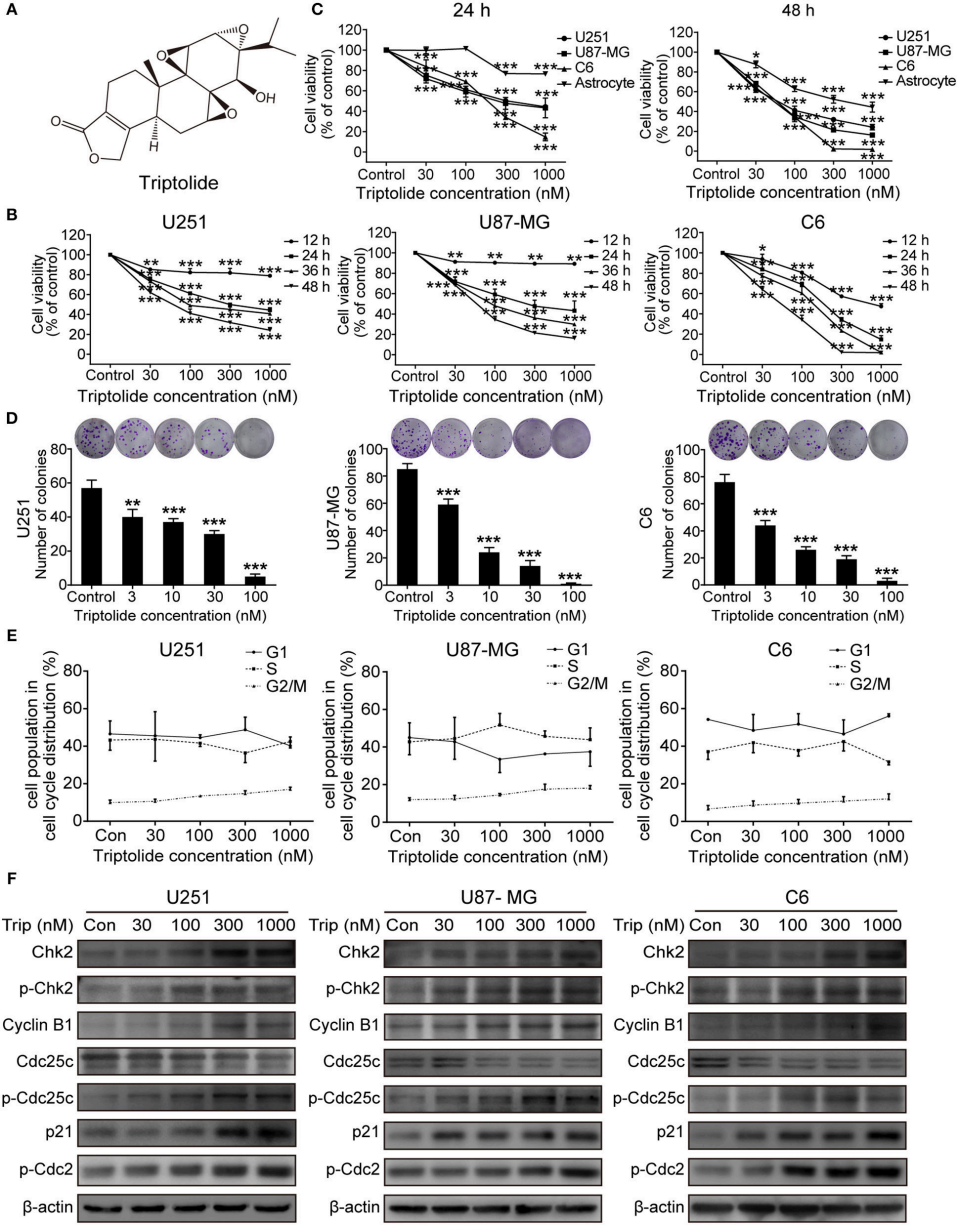
Triptolide (Trip) inhibited the proliferation of glioma cells and arrested cells in the G2/M phase. **(A)** Chemical structure of triptolide. **(B)** U251, U87-MG and C6 cells were treated with the indicated concentrations of triptolide or vehicle (DMSO) for 12–48 h, and the cell viability was quantified by a CCK8 assay. **(C)** Three glioma cell lines and primary cultured astrocyte cells were treated with the indicated concentrations of triptolide or vehicle for 24 and 48 h, and the cell viability was measured by a CCK8 assay. **(D)** Three glioma cell lines were treated with the indicated concentrations of triptolide or vehicle for 10 days. Cell colonies were stained with crystal violet, and the colonies were quantified (cell number >50). **(E)** U251, U87-MG, and C6 cells were treated with triptolide for 24 h and stained with PI. The PI staining data were quantified as the percentage of cells in the G1, S, and G2/M phases. **(F)** U251, U87-MG, and C6 cells were treated with triptolide for 24 h. Whole-cell lysates were separated by SDS-PAGE, and then, immunoblotting was performed using the indicated antibodies. The data represent 3 independent experiments. The graphs indicate the means ± SD of data obtained from 3 independent experiments. ^*^P < 0.05, ^**^P < 0.01, ^***^P < 0.001, significantly different than the untreated control group.

To assess the cytotoxic effect of the triptolide (Figure 1A) treatment on glioma cells, a CCK8 assay and colony formation assay were used. As shown in Figure 1B, the CCK8 assay showed that triptolide significantly reduced the cell viability in the U251, U87MG, and C6 cells after incubation for 12 h and inhibited the growth of glioma cells in a time- and dose-dependent manner with IC50 values of 170–400 nM (24 h) and 50–80 nM (48 h) (Table S1). However, the inhibitory effect of triptolide on primary cultured astrocyte cells was not significant with IC50 values of 6835.2 nM and 431.4 nM at 24 and 48 h, respectively (Figure 1C and Table S1). Moreover, triptolide induced morphological alterations in the glioma cells (Figure S1A) and dramatically inhibited colony formation (Figure 1D). These results suggest that compared to primary cultured astrocyte cells, the glioma cells were especially sensitive to the triptolide treatment.

Defects in cell-cycle progression can lead to cell death or contribute to cancer progression (26). Therefore, cell cycle progression was analyzed. The cell cycle distribution analysis showed that treatment with a certain concentration of triptolide for 24 h increased the number of cells in the G2/M phase (Figure S1B and Figure 1E). Furthermore, the western blotting assays showed changes in the expression of cell-cycle-regulated proteins. As shown in Figure 1F, triptolide upregulated the expression of CyclinB1, phospho-Cdc2 (p-Cdc2), Chk2, phospho-Chk2 (p-Chk2), phospho-Cdc25C (p-Cdc25C), and p21 and downregulated the expression of Cdc25C. Thus, triptolide inhibited glioma cell growth by inducing cell death and cell cycle arrest at the G2/M phase.

### Triptolide Triggers Apoptosis in Glioma Cells

**Figure 2:**
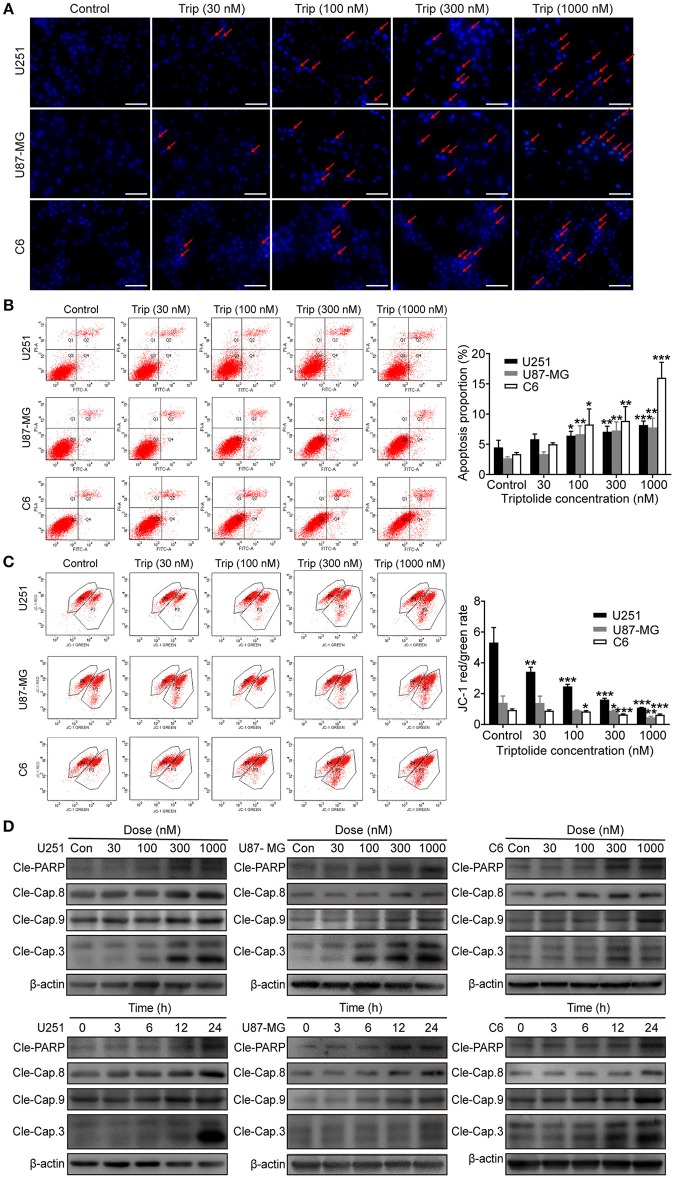
Triptolide induced apoptosis in glioma cells. U251, U87-MG, and C6 cells were treated with the indicated concentrations of triptolide or vehicle for 24 h. **(A)** Cells were fixed and stained with Hoechst 33342 and observed under a fluorescence microscope. **(B)** Apoptosis was analyzed by FACS using an Annexin V-FITC/PI cell apoptosis kit. Representative results from 3 independent experiments are presented. The data are presented as the means ± SD (*n* = 3). (**C)** MMP was assessed by FACS based on fluorescent mitochondria. The data are presented as the means ± SD (*n* = 3). (**D)** Cells were treated with the indicated concentrations of triptolide for 24 h or incubated with triptolide (300 nM) for different durations. Whole-cell lysates were separated by SDS-PAGE, and then, immunoblotting was performed using the indicated antibodies. ^*^P < 0.05, ^**^P < 0.01, ^***^P < 0.001, significantly different compared with the untreated control group.

**Figure 3:**
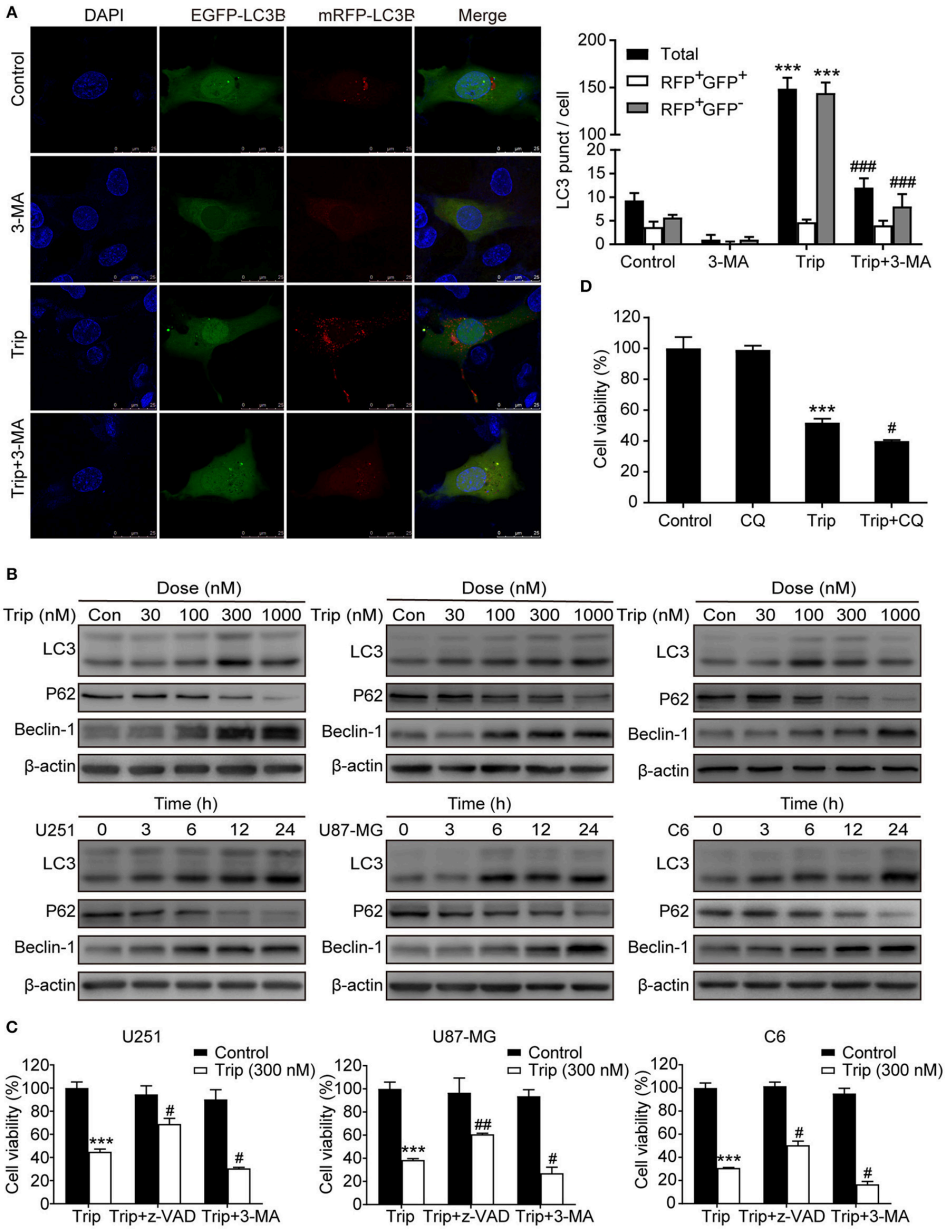
Triptolide induced protective autophagy in glioma cells. **(A)** Cells stably expressing mRFP-EGFP-LC3B were pretreated with 3-MA (3 mM) for 2 h, followed by treatment with triptolide (150 nM) for an additional 24 h. Representative fluorescent images were photographed by laser scanning confocal microscopy. Scale bars = 25 μm. **(B)** Cells were treated with the indicated concentrations of triptolide for 24 h or incubated with triptolide (300 nM) for 0–24 h, and the autophagy-related proteins LC3, p62, and Beclin-1 were analyzed by western blotting. **(C)** U251, U87-MG, and C6 cells were pretreated with 3-MA or z-VAD (30 μM) for 2 h, followed by treatment with triptolide (300 nM) for an additional 24 h. Cell viability was determined by a CCK8 assay. **(D)** U251 cells were pretreated with CQ (20 μM) for 2 h, followed by treatment with triptolide (300 nM) for an additional 24 h. Cell viability was determined by a CCK8 assay. The data are presented as the means ± SD (*n* = 3). ^***^P < 0.001, significantly different compared with the untreated control group. ^#^*P* < 0.05, ^##^*P* < 0.01, ^###^*P* < 0.001 significantly different compared with the triptolide treatment group.

To obtain a deeper understanding of the molecular mechanisms underlying the inhibitory effect of triptolide on glioma cells, we further examined the effect of triptolide on apoptosis. To analyze apoptosis in the glioma cells, Hoechst 33342 staining, an Annexin V/PI assay, an MMP assay and a western blotting assay were employed. As shown in Figure 2A, the glioma cells treated with triptolide for 24 h showed some typical apoptotic morphological changes as indicated by the red arrows, such as cell shrinkage, chromatin condensation, and nuclear fragmentation, as revealed by the Hoechst 33342 staining. Exposure to 30–1000 nM triptolide increased the rate of apoptosis in a concentration-dependent manner as assessed by flow cytometry using an Annexin V/PI assay (Figure 2B). We also examined the triptolide-induced changes in the MMP. The triptolide treatment induced the conversion of red fluorescence to green fluorescence, indicating a decrease in MMP as revealed by flow cytometry after JC-1 staining (Figure 2C). The western blotting analysis further corroborated these results. As shown in Figure 2D, the whole-cell extracts of the treated cells exhibited high levels of the active forms of PARP, caspase-3, caspase-8 and caspase-9, which increased in a time- and dose-dependent manner. Moreover, z-VAD (a caspase inhibitor) could reverse a proportion of cell death (Figure 3C). These results confirm that triptolide triggers apoptosis in glioma cells.

### Triptolide Activates Autophagy as a Protective Mechanism in Glioma Cells

To determine whether autophagy plays a role in triptolide-treated cells, we analyzed the autophagic activity in the glioma cells. The U251 cell lines were transiently transfected with the mRFP-EGFP-LC3 construct 24 h prior to the triptolide treatment. Following the treatment, the cells were fixed and observed by confocal microscopy. As shown in Figure 3A, significant fluorescence signals of the mostly autophagy-specific protein LC3 were observed in the triptolide-treated cells with puncta accumulation. To further confirm that triptolide induces autophagy in glioma cells, we determined the expression of LC3B, p62 and Beclin-1, which are important autophagy-related proteins, using western blotting. As shown in Figure 3B, the triptolide treatment significantly augmented the expression levels of LC3B and Beclin-1 and dramatically reduced the expression level of p62 in a dose- and time-dependent manner in the glioma cells. In cancer cells, autophagy can have onco-suppressive or tumor-supporting effects (27). Therefore, elucidating the function of triptolide-induced autophagy in cell death is essential. Thus, 3-MA, which is an inhibitor of the formation of autophagosomes, was used to inhibit the cellular autophagic pathway. As shown in Figure 3C, the treatment with 3-MA increased triptolide-induced cell death. In addition, we also used chloroquine diphosphate salt (CQ) to inhibit the degradation of autophagy by increasing the pH of the lysosome in U251 cells. As shown in Figure 3D, cell viability was decreased compared with that of triptolide treatment alone. Taken together, these results suggest that the triptolide-treated cells exhibited higher basal autophagy activity as a protective mechanism against the effects of triptolide in glioma cells.

### Triptolide-Induced Autophagy and Apoptosis can Cross-Inhibit Each Other

**Figure 4:**
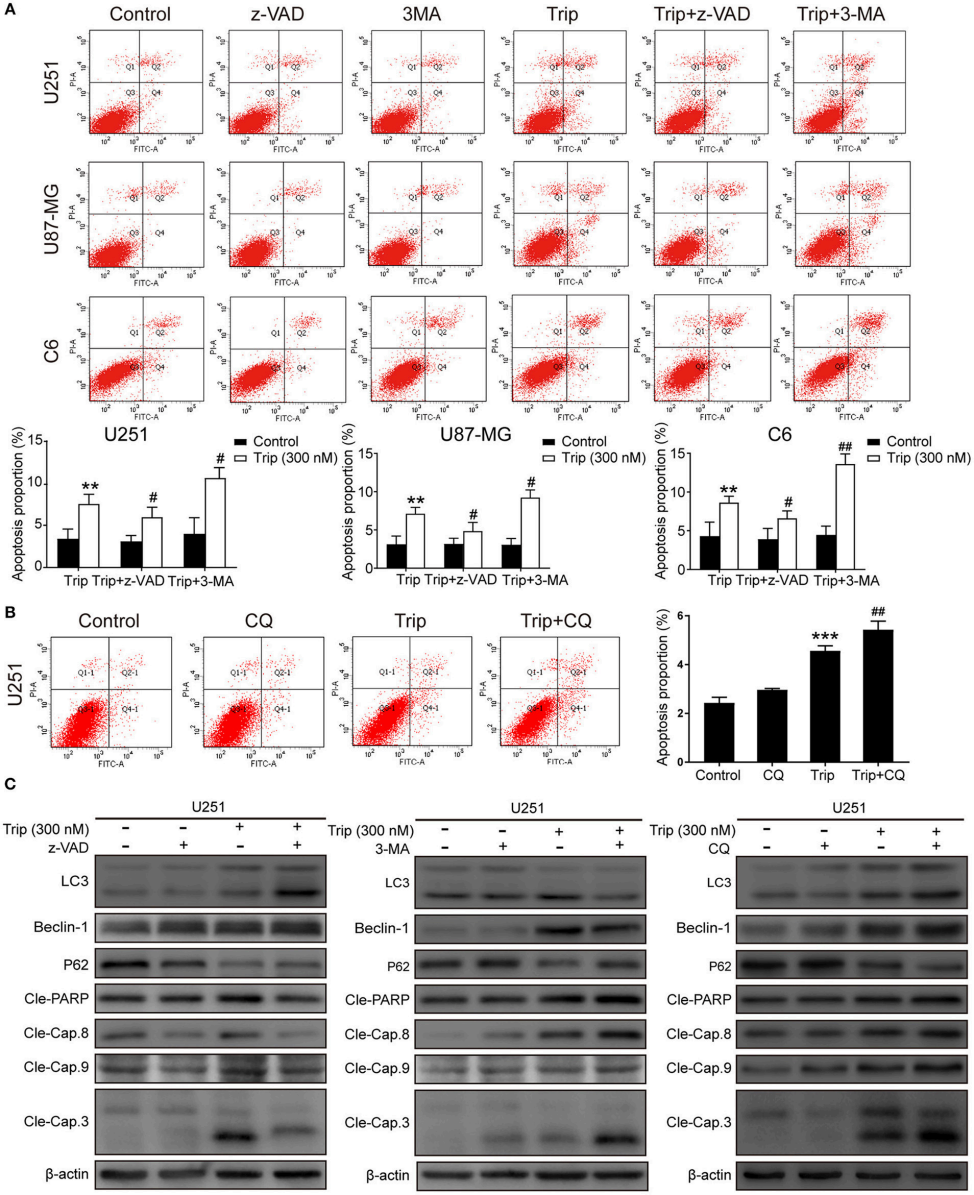
Autophagy and apoptosis induced by triptolide can cross-inhibit each other. **(A)** U251, U87-MG, and C6 cells were pretreated with 3-MA or z-VAD for 2 h, followed by treatment with triptolide (300 nM) for an additional 24 h. Apoptosis was analyzed by FACS using an Annexin V-FITC/PI cell apoptosis kit. Representative results from 3 independent experiments are presented. The data are presented as the means ± SD (*n* = 3). (**B)** U251 cells were pretreated with CQ for 2 h, followed by treatment with triptolide (300 nM) for an additional 24 h. Apoptosis was analyzed by FACS using an Annexin V-FITC/PI cell apoptosis kit. **(C)** U251 cells were pretreated with 3-MA or z-VAD or CQ for 2 h, followed by treatment with triptolide (300 nM) for an additional 24 h. Whole-cell lysates were separated by SDS-PAGE, and then, immunoblotting was performed using the indicated antibodies. ^**^P < 0.01, ^***^P < 0.001, significantly different compared with the untreated control group. ^#^*P* < 0.05, ^##^*P* < 0.01, significantly different compared with the triptolide treatment group.

Autophagy and apoptosis are two processes that determine cell fate by regulating the balance between survival and death. We further explored the relationship between autophagy and apoptosis induced by triptolide. As shown in Figures 4A, B, the combination of triptolide and 3-MA or CQ markedly increased the proportion of apoptotic cells compared to that observed after the triptolide treatment alone. Consistently, the autophagy inhibition by 3-MA or CQ also led to the activation of caspases, including caspase-3, caspase-8, and caspase-9, and the resultant cleavage of PARP. The pretreatment with z-VAD also dramatically increased the expression levels of the autophagy-related proteins LC3B and Beclin-1 and decreased the level of p62 (Figure 4C). Thus, we believe that both apoptosis and autophagy are preserved in glioma cells following triptolide treatment and that these two processes tend to mutually restrain each other.

### ROS Production and Subsequent JNK Activation and Akt/mTOR Signaling Pathway Inhibition Play an Essential Role in Triptolide-Induced Autophagy and Cell Death

**Figure 5:**
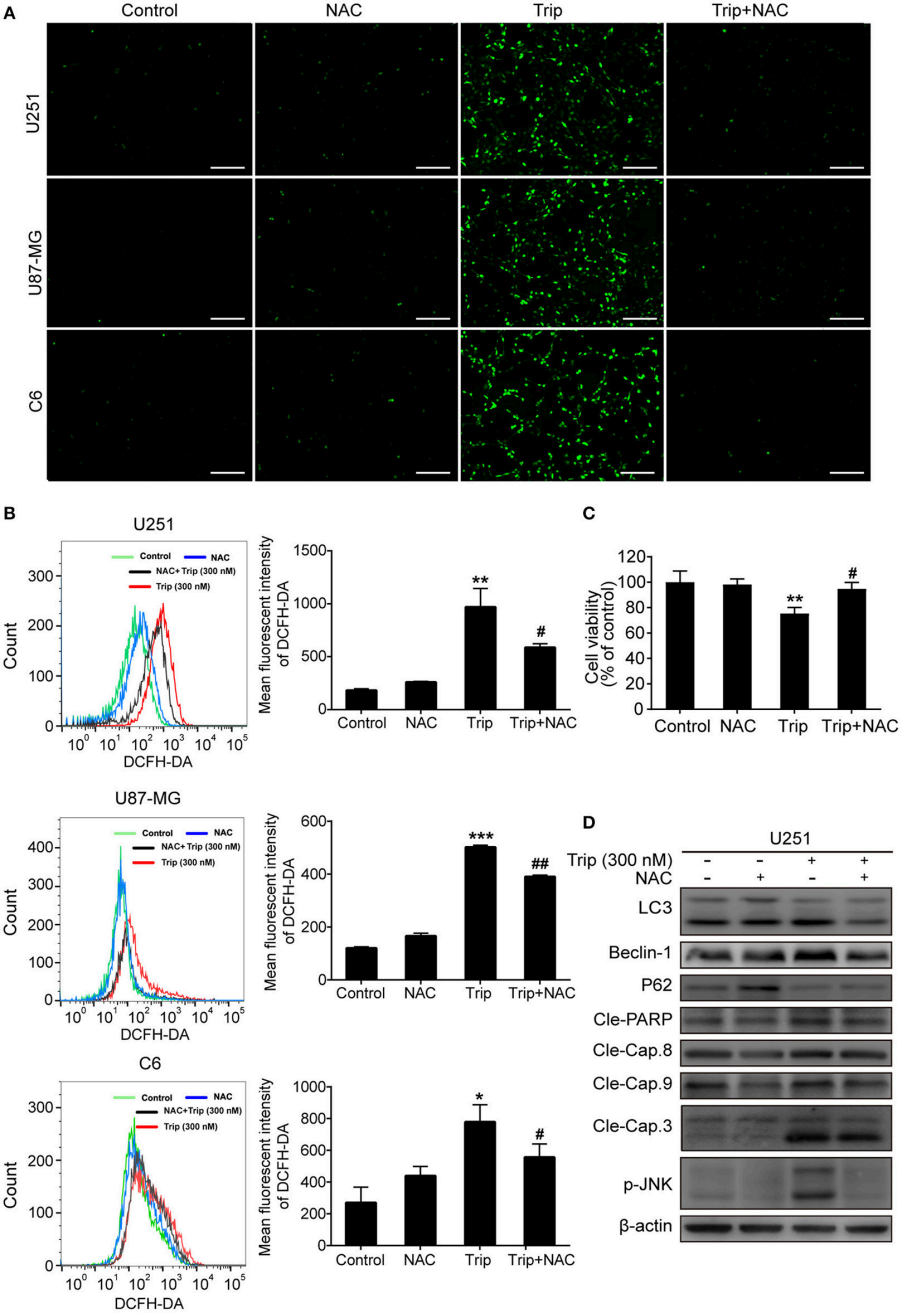
ROS production played an essential role in triptolide-induced autophagy, apoptosis, and cell death. (**A–D)** U251, U87-MG, and C6 cells were treated with triptolide (300 nM) in the absence or presence of NAC (5 mM) for 12 h. (**A)** After incubation, the cells were stained with 10 μM DCFH-DA at 37°C in the dark for 20 min and observed under a fluorescence microscope. **(B)** The ROS level was tested by flow cytometry, and the mean fluorescence intensity of DCFH-DA was determined. U251 cells were pretreated with NAC for 2 h, followed by treatment with triptolide (300 nM) for an additional 12 h. (**C)** The viability of the cells was measured by a CCK8 assay. (**D)** Whole-cell lysates were separated by SDS-PAGE, and then, immunoblotting was performed using the indicated antibodies. Representative results from 3 independent experiments are presented. ^*^P < 0.05, ^**^P < 0.01, ^***^P < 0.001, significantly different compared with the untreated control group. ^#^*P* < 0.05, ^##^*P* < 0.01, significantly different compared with the triptolide treatment group.

The molecular regulatory processes of autophagy and apoptosis are intertwined at different levels (27). Thus, we subsequently investigated the underlying upstream regulatory mechanisms leading to the induction of autophagy and apoptosis by triptolide. As intracellular ROS play a critical role in different types of cell survival (28), we investigated whether triptolide increases ROS production in glioma cells. Thus, N-acetylcysteine (NAC), which is usually considered a regulator of ROS release, was used to alter ROS production. The ROS level was detected using the fluorescent probe DCFH-DA by fluorescence microscopy and flow cytometry. As shown in Figures 5A,B, clear increases in the fluorescent signals and the mean fluorescence intensity of DCFH-DA were observed in the treated glioma cells. Accumulated ROS can be scavenged by NAC; thus, to investigate whether ROS production was involved in the triptolide-induced autophagy, apoptosis, and cell death, we used this pharmacological inhibitor of ROS. The pretreatment of U251 cells with NAC significantly reduced triptolide-induced apoptosis and autophagy as determined by a western blotting assay (Figure 5D) and cell death as determined by a CCK-8 assay (Figure 5C). These data indicate that ROS accumulation is necessary for cell autophagy and apoptosis induced by triptolide in glioma cells.

**Figure 6:**
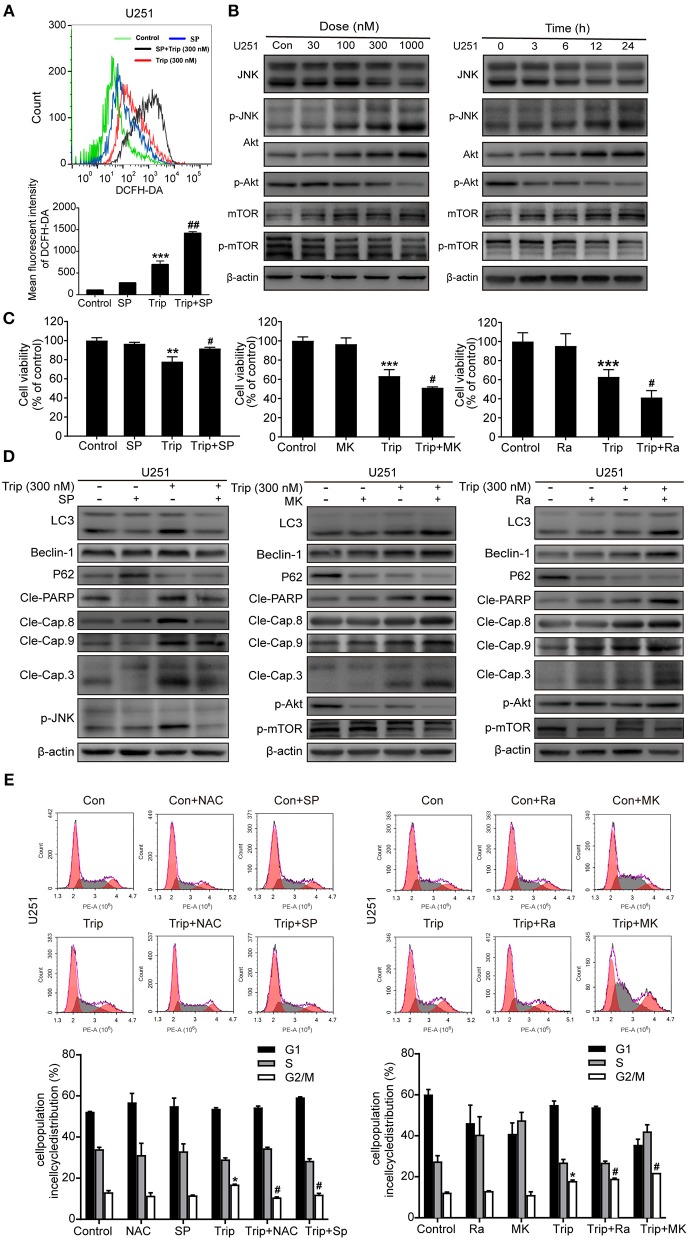
Triptolide induced autophagy and apoptosis via the activation of the ROS/JNK and inhibition of the Akt/mTOR signaling pathways. **(A)** U251 cells were pretreated with SP (40 μM) for 2 h, followed by treatment with triptolide (300 nM) for an additional 12 h. The ROS level was determined by flow cytometry. The data are presented as the means ± SD (*n* = 3). **(B)** After treating the U251 cells with various concentrations of triptolide for 24 h or 300 nM triptolide for different durations, the phosphorylation of the JNK and Akt/mTOR signaling pathways was detected by a western blot analysis. Cells were pretreated with MK (2 μM) for 5 h or Ra (1 μM) or SP for 2 h and treated with 300 nM triptolide for 24 h. **(C)** Cell viability was measured by a CCK8 assay. The data are presented as the means ± SD (*n* = 3). **(D)** The apoptosis-related proteins, autophagy-related proteins and phosphorylation levels of the JNK and Akt/mTOR signaling pathways were analyzed by western blotting. **(E)** Glioma cells were treated with 300 nM triptolide in the presence or absence of NAC, SP, MK, and Ra for 24 h, and the cell cycle was evaluated by flow cytometry. The cell cycle distribution is presented in histograms. The data are presented as the means ± SD (*n* = 3). ^*^P < 0.05, ^**^P < 0.01, ^***^P < 0.001, significantly different compared with the untreated control group. ^#^*P* < 0.05, ^##^*P* < 0.01, significantly different compared with the triptolide treatment group.

The increases in ROS can, in turn, activate the redox-sensitive JNK signaling pathway, which plays a vital role in apoptosis (28). Thus, we examined JNK activation induced by triptolide. As shown in Figure 6B, the triptolide treatment induced considerable activation of JNK in the U251 cells. Notably, this prolonged JNK activation was eliminated in the cells pretreated with NAC (Figure 6D), while the pretreatment with SP600125 (SP), which is usually considered an inhibitor of JNK, increased the generation of ROS following the triptolide treatment (Figure 6A), indicating that ROS generation is essential for triptolide-induced JNK activation. The Akt/mToR signaling pathway, which is among the major regulatory pathways of cell survival, was also investigated. As shown in Figure 5B, the triptolide treatment blocked the inhibitory activity of Akt and mTOR. Taken together, these results indicate that triptolide can promote the activation of JNK, which is related to the production of ROS, and inhibit the activity of Akt and mTOR.

We further investigated the roles of JNK and the Akt/mTOR signaling pathway. As shown in Figures 6C,D, the cell viability was enhanced and the levels of autophagy and apoptosis were reduced after the SP treatment compared with those observed following the treatment with triptolide alone, while MK2206 (MK) and rapamycin (Ra), which are usually considered inhibitors of Akt and mTOR, had the opposite effect on the triptolide-treated U251 cells. In the cells pretreated with MK and Ra, the expression levels of apoptosis-related and autophagy-related proteins were increased, and cell death was also increased. In addition, MK notably inhibited the activation of Akt and mTOR, while Ra did not suppress the activation of Akt. Moreover, NAC, SP, MK, and Ra all altered the cell cycle distribution mediated by triptolide (Figure 6E). Thus, these results suggest that prolonged JNK activation following the enhanced production of ROS and the inhibition of the Akt/mTOR signaling pathway contribute to triptolide-induced autophagy and apoptosis.

### Triptolide Inhibits the Growth of Gliomas in a Mouse Orthotopic Xenograft Model *in Vivo*

**Figure 7:**
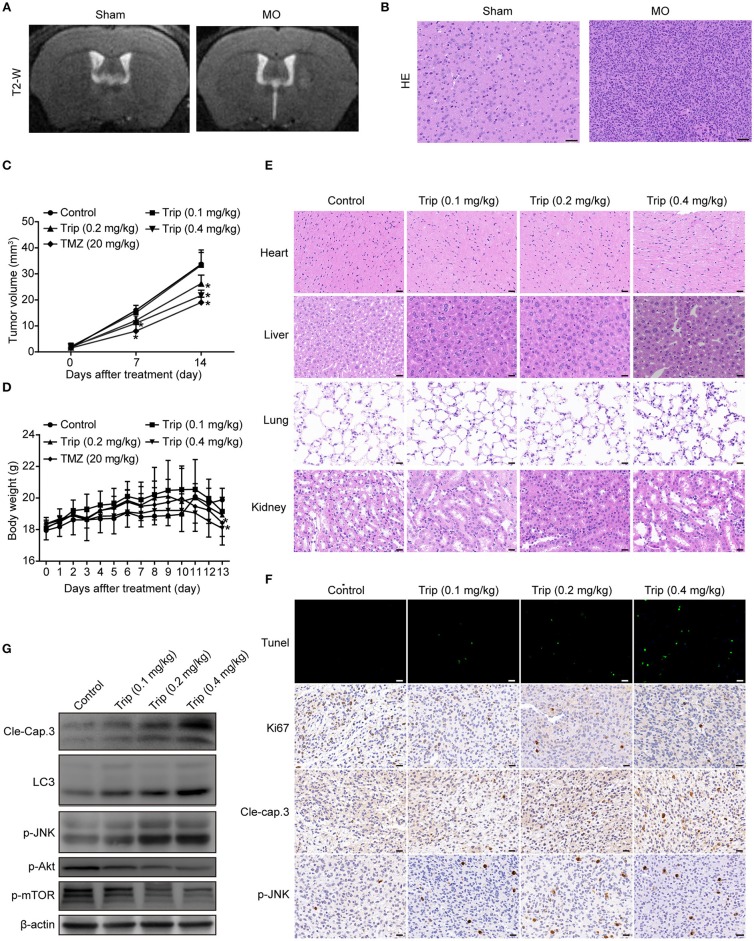
Triptolide inhibited tumor growth in a U251 orthotopic xenograft model. **(A)** One week after inoculation, T2-weighted MRI images of gliomas were obtained with a 7.0 T MRI scanner. **(B)** H&E staining of gliomas in sham and model mice. Scale bars = 50 μm. **(C)** Tumor volumes were determined by MRI at 0, 7, and 14 days after treatment in the vehicle control mice, triptolide-treated mice and TMZ-treated mice. The bars represent the means ± SD. **(D)** Body weight changes in mice during 13 days of exposure. The bars represent the means ± SD. **(E)** H&E staining of important organs in the vehicle control mice and triptolide-treated mice. Scale bars = 20 μm. **(F)** TUNEL measurement of apoptosis and immunohistochemical staining of tumor specimens. Scale bars = 20 μm. **(G)** Western blot quantification of cleaved caspase-3, LC3B, phospho-JNK, phospho-Akt, and phospho-mTOR expression. ^*^P < 0.05, significantly different compared with the vehicle control group.

To evaluate the antitumor activity of triptolide *in vivo*, human glioma orthotopic xenografts were established by injecting ~3.5 × 10^4^ U251 cells into the right striatal area of nude mice. At 7 days after the injection, the tumor-bearing mice were observed via T2-weighted MRI images. As shown in Figure 7A, the T2-weighted images showed a strong signal in the right striatum in the model group. The hematoxylin and eosin (H&E) staining revealed the general characteristics of malignancy, including a significantly increased cell density, distinct nuclear atypia and common mitotic figures, only in the model group (Figure 7B). These results suggest that we successfully established a human glioma orthotopic xenograft model. Then, the tumor-bearing mice were randomly distributed into the vehicle control and treatment groups and intraperitoneally treated every other day with either 0 mg/kg (vehicle control group) or 0.1 mg/kg, 0.2 mg/kg, or 0.4 mg/kg triptolide (treatment groups) for a total of 7 treatments. TMZ, which served as a positive control, was intraperitoneally administered every other day at a dose of 20 mg/kg. As depicted in Figure 7C, 0.2 and 0.4 mg/kg triptolide effectively inhibited the growth of the tumors *in vivo*. Moreover, the mice treated with 0.2 mg/kg triptolide had no weight loss, while the body weight of the 0.4 mg/kg triptolide-treated mice slightly decreased (Figure 7D). The results of the H&E staining showed that compared with the vehicle control group, the triptolide-treated groups showed no obvious changes, highlighting the low toxicity of triptolide at a dose of 0.4 mg/kg (Figure 7E).

To further investigate the mechanism of the inhibitory effect of triptolide *in vivo*, the tumor tissues were analyzed by a TUNEL assay, immunohistochemistry with antibodies against Ki67, cleaved caspase-3 and p-JNK, and western blotting with primary antibodies against cleaved caspase-3, LC3, p-JNK, p-Akt and p-mTOR. As shown in Figures 7F,G, Figure S2 , compared to the control treatment, the triptolide treatment caused significant increases in TUNEL-positive cells and cleaved caspase-3, LC3, and p-JNK immunoreactivity; in contrast, compared with the control group, triptolide caused significant decreases in Ki67, p-Akt, and p-mTOR. These findings suggest that the antitumor mechanism of triptolide is associated with the induction of apoptosis and autophagy through the ROS/JNK and Akt/mTOR signaling pathways.

## Discussion

Autophagy is a caspase-independent cell death pathway involving a conserved lysosomal degradation process crucial for maintaining homeostasis and development (29). Autophagy plays a key role in the antitumor mechanisms of chemotherapeutic agents and may be an important cytoprotective mechanism (30). Apoptosis, which is a tightly regulated form of programmed cell death, is activated to efficiently eliminate dysfunctional cells. Apoptosis in tumor cells induced by chemotherapeutic agents is an important mechanism in the treatment of tumors (31). As the two most prominent forms of developmental cell death, autophagy and apoptosis engage in crosstalk as reported in many studies; for example, FOXO3a, which is an autophagy-regulating transcription factor, stimulates the transcription of the pro-apoptotic gene BBC3/PUMA to cause apoptosis sensitization after the inhibition of autophagy (32). Furthermore, ATG3, which is a protein critical for autophagosome formation, triggers apoptosis when overexpressed in adherent cells (33). A prior study demonstrated that triptolide inhibits the proliferation of malignant glioma cells (16). Based on the crosstalk findings, apoptosis and autophagy are likely involved in inhibiting growth in triptolide-treated glioma cells. Here, we examined triptolide-induced apoptosis and autophagy and revealed a novel mechanism by which autophagy inhibits apoptosis and protects glioma cells against cell death.

To verify the effects of triptolide on glioma cells, cell death and cell cycle progression were first analyzed. We found that triptolide inhibited the proliferation of glioma cells and arrested the cell cycle in the G2/M phase. In mammalian cells, cell cycle progression is regulated by a group of CDKs and their regulatory subunits. CyclinB1, Cdc2 (also known as cyclin-dependent kinase 1, CDK1), Cdc25c, Chk2, and p21 play a crucial role in the G2 checkpoint. The concentration of cyclinB1 displays periodic behavior and reaches a critical threshold at the end of the G2 threshold. Before reaching this threshold, cyclinB1 interacts primarily with cdc2 to regulate the G2-M transition, and the cyclinB1/Cdc2 complex is maintained in an inactive state (34, 35). Cdc25 phosphatases are key regulators of the eukaryotic cell cycle, and at the onset of mitosis, Cdc25c completely activates the Cdc2-cyclinB1 complex in the nucleus (36). Surprisingly, in this study, the expression of p21 was also increased by the triptolide treatment. P21 (a CDK inhibitor) is a key negative regulator of cell cycle progression that mediates cell cycle arrest at the G1 or G2 phase in a p53-dependent or p53-independent manner (37). However, whether p53 is involved in the triptolide-induced cell cycle arrest needs to be further investigated.

To clarify the role of autophagy and the connections between autophagy and apoptosis in triptolide-induced glioma cells, the levels of apoptosis and autophagy were examined. The extrinsic apoptotic pathway is also known as the death receptor pathway in which caspase-8, caspase-3 and a downstream cascade of effectors are activated. The other apoptotic pathway is the intrinsic pathway, which is also called the mitochondrial pathway, leading to decreased MMP and the activation of caspase-9 and additional caspase molecules, such as caspase-3, caspase-6 and caspase-7. The two pathways are accompanied by typical morphological changes (38). We found that triptolide induced apoptosis in glioma cells. However, further studies will be needed to identify specific apoptosis pathway in glioma cells treated by triptolide. Furthermore, the z-VAD treatment effectively suppressed triptolide-induced cell death. As LC3 is widely used as a marker protein to assess autophagosome formation, Beclin1 is an upstream molecule required for autophagosome formation, and p62 is a ubiquitin-binding receptor protein that is scavenged and degraded by autophagy, we chose to use LC3, Beclin1, and p62 to examine the induction of autophagy (39). The induction of autophagy with triptolide was enhanced, and cell death was increased in response to the 3-MA treatment, suggesting that triptolide induced apoptosis and autophagy in glioma cells and that autophagy may exert a protective effect. To further investigate the crosstalk between apoptosis and autophagy induced by triptolide, 3-MA and z-VAD were used. In this study, we provide the first strong evidence suggesting that triptolide-induced autophagy limits apoptosis, which is beneficial to tumor cell survival in glioma cells. Similar results have been obtained with other tumor cells, including colorectal cancer cells and prostate cancer cells, in which autophagy inhibition mediated apoptosis sensitization, suggesting that autophagy favors the survival of tumor cells and that the processes of apoptosis and autophagy can cross-inhibit each other under certain environmental conditions (40, 41).

Mounting evidence suggests that multifaceted signaling molecules released by mitochondria from the electron transport chain are involved in the development and progression of many types of diseases. For example, the excessive accumulation of ROS, which are included among these signaling molecules, can promote cancer (21, 42). The present findings provide direct evidence that triptolide enhances ROS generation in glioma cells, which, in turn, is responsible for the apoptosis and autophagy induced by triptolide in these cells. This finding is supported by the fact that the ROS scavenger NAC is able to prevent most of these effects. As members of the MAPK pathways, which are some of the numerous downstream cascades of the ROS signaling pathway, JNKs can function as pro-apoptotic kinases that play a critical role in both the extrinsic and intrinsic apoptotic pathways (22, 43). Therefore, we speculated that ROS activated JNK and used the regulators SP, MK and Ra to examine the function of JNK and the Akt/mTOR signaling pathways in triptolide-treated glioma cells. Our results showed that compared to after the treatment with triptolide alone, the cell viability was enhanced, and autophagy and apoptosis were reduced after the SP treatment. MK and Ra produced the opposite effects. These findings suggest that the ROS/JNK signaling pathways were activated, but the Akt/mTOR signaling pathway was inhibited in the triptolide-treated glioma cells. Furthermore, our results showed that NAC, SP, MK, and Ra could affect cell cycle progression; NAC and SP attenuated the triptolide-induced G2/M phase arrest, while MK and Ra greatly increased the G2/M phase arrest in the glioma cells.

In our *in vivo* study, the 0.2 mg/kg triptolide-treated mice and 0.4 mg/kg triptolide-treated mice demonstrated significantly reduced tumor volumes; in the 0.4 mg/kg triptolide-treated mice, this effect was accompanied by slight weight loss without major visible toxicity, revealing that triptolide could inhibit tumor growth and that high doses of triptolide may have few side effects. In additional, triptolide has not been approved for clinical trials because of its poor water solubility and toxicity. For triptolide to be used clinically in the future, the optimal doses of triptolide need to be cautiously determined.

Collectively, our results indicate that apoptosis and autophagy are critical processes in glioma cells following triptolide treatment. Triptolide can induce apoptosis and autophagy in glioma cells, which has not been previously reported. Furthermore, the role of triptolide-induced autophagy is protective. More importantly, the apoptosis and autophagy induced by triptolide can cross-inhibit each other, and triptolide regulates apoptosis and autophagy by activating the ROS/JNK and suppressing the Akt/mTOR signaling pathways. However, several critical questions remain unanswered. For example, what are the possible targets of ROS that could play a key role in the Akt/mTOR signaling pathway? What are the interactions between autophagy and apoptosis? Answering these questions could be important for identifying the precise molecular mechanism underlying the observed effects.

**Figure 8:**
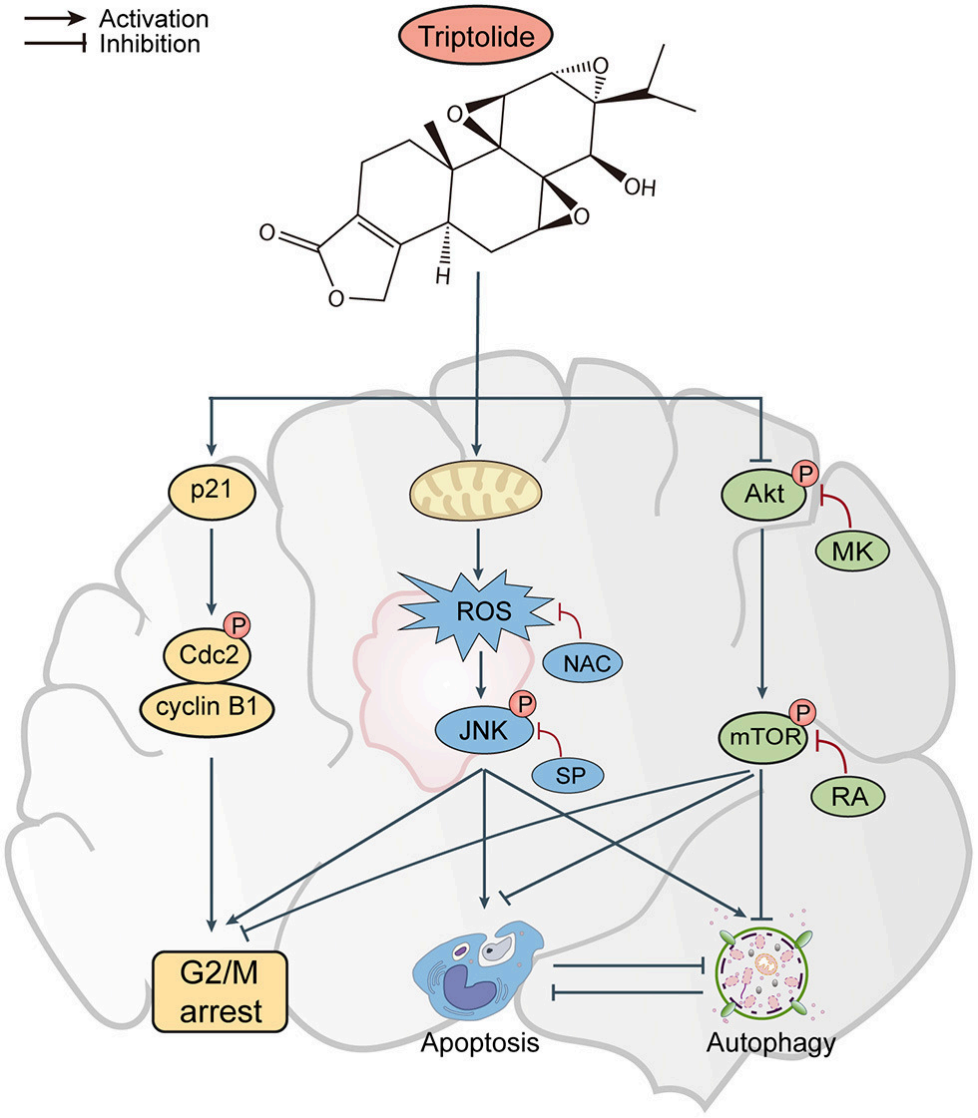
Proposed mechanism of triptolide-induced apoptosis and autophagy in glioma cells.

In conclusion, the results of our *in vitro* and *in vivo* studies demonstrate that triptolide can induce apoptosis and autophagy through the activation of the ROS/JNK and the inhibition of the Akt/mTOR signaling pathways. We further demonstrated that the role of autophagy is protective and that apoptosis and autophagy can cross-inhibit each other (Figure 8). These findings suggest that triptolide-induced autophagy is a potential mechanism underlying the effects of triptolide in glioma cells. However, the precise molecular mechanism needs to be characterized in the future as such characterization could be important for further investigations of the antitumor effect of triptolide in glioma cells.

## Data Availability

All datasets generated for this study are included in the manuscript and/or the Supplementary Files.

## Ethics Statement

This study was carried out in accordance with the recommendations of Animal Experiments and Experimental Animal Welfare Committee of Capital Medical University. The protocol was approved by the Animal Experiments and Experimental Animal Welfare Committee of Capital Medical University.

## Author Contributions

XL, XW, and WG contributed to the conception and design of the study. XL, PZ, and YZ performed the experiments. XL, PZ, and LW analyzed the data. XL, XW, and WG drafted the manuscript. All authors contributed to the manuscript writing and approved the final manuscript.

### Conflict of Interest Statement

The authors declare that the research was conducted in the absence of any commercial or financial relationships that could be construed as a potential conflict of interest.

## References

[1] Wen PY Reardon DA. Neuro-oncology in 2015: Progress in glioma diagnosis, classification and treatment. Nat Rev Neurol. (2016) 12:69–70. 10.1038/nrneurol.2015.242 26782337

[2] Ghinda DC Wu JS Duncan NW Northoff G. How much is enough-Can resting state fMRI provide a demarcation for neurosurgical resection in glioma? Neurosci Biobehav Rev. (2018) 84:245–61. 10.1016/j.neubiorev.2017.11.019 29198588

[3] Lapointe S Perry A Butowski NA. Primary brain tumours in adults. Lancet. (2018) 392:432–46. 10.1016/S0140-6736(18)30990-5 30060998

[4] Bi Y Li H Yi D Bai Y Zhong S Liu Q. beta-catenin contributes to cordycepin-induced MGMT inhibition and reduction of temozolomide resistance in glioma cells by increasing intracellular reactive oxygen species. Cancer Lett. (2018) 435:66–79. 10.1016/j.canlet.2018.07.040 30081068

[5] Zhou ZL Yang YX Ding J Li YC Miao ZH. Triptolide: structural modifications, structure-activity relationships, bioactivities, clinical development and mechanisms. Nat Prod Rep. (2012) 29:457–75. 10.1039/c2np00088a 22270059

[6] Titov DV Gilman B He QL Bhat S Low WK Dang YJ. XPB, a subunit of TFIIH, is a target of the natural product triptolide. Nat Chem Biol. (2011) 7:182–8. 10.1038/Nchembio.522 21278739 PMC3622543

[7] Zheng L Jia J Dai H Wan L Liu J Hu L. Triptolide-Assisted Phosphorylation of p53 Suppresses Inflammation-Induced NF-kappaB Survival Pathways in Cancer Cells. Mol Cell Biol. (2017) 37:e00149–17. 10.1128/MCB.00149-17 28533220 PMC5514447

[8] Lue Y Sinha Hikim AP Wang C Leung A Baravarian S Reutrakul V. Triptolide: a potential male contraceptive. J Androl. (1998) 19:479–86 9733151

[9] Ling D Xia H Park W Hackett MJ Song C Na K. pH-Sensitive nanoformulated triptolide as a targeted therapeutic strategy for hepatocellular carcinoma. Acs Nano. (2014) 8:8027–39. 10.1021/nn502074x 25093274

[10] Chen JX Qiao YT Tang B Chen G Liu XF Yang BY. Modulation of salmonella tumor-colonization and intratumoral anti-angiogenesis by triptolide and its mechanism. Theranostics. (2017) 7:2250–60. 10.7150/thno.18816 28740548 PMC5505057

[11] Corson TW Crews CM. Molecular understanding and modern application of traditional medicines: triumphs and trials. Cell. (2007) 130:769–74. 10.1016/j.cell.2007.08.021 17803898 PMC2507744

[12] Yang A Qin S Schulte BA Ethier SP Tew KD Wang GY. MYC inhibition depletes cancer stem-like cells in triple-negative breast cancer. Cancer Res. (2017) 77:6641–50. 10.1158/0008-5472.CAN-16-3452 28951456 PMC5712265

[13] Ding BY Wahid MA Wang ZJ Xie C Thakkar A Prabhu S. Triptolide and celastrol loaded silk fibroin nanoparticles show synergistic effect against human pancreatic cancer cells. Nanoscale. (2017) 9:11739–53. 10.1039/c7nr03016a 28782773 PMC5648537

[14] Han Y Huang W Liu J Liu D Cui Y Huang R. Triptolide inhibits the AR signaling pathway to suppress the proliferation of enzalutamide resistant prostate cancer cells. Theranostics. (2017) 7:1914–27. 10.7150/thno.17852 28638477 PMC5479278

[15] Dauer P Zhao X Gupta VK Sharma N Kesh K Gnamlin P. Inactivation of cancer-associated-fibroblasts disrupts oncogenic signaling in pancreatic cancer cells and promotes its regression. Cancer Res. (2018) 78:1321–33. 10.1158/0008-5472.CAN-17-2320 29259015 PMC5935584

[16] Zhang H Zhu W Su X Wu S Lin Y Li J. Triptolide inhibits proliferation and invasion of malignant glioma cells. J Neurooncol. (2012) 109:53–62. 10.1007/s11060-012-0885-5 22562416

[17] Sarosiek KA Fraser C Muthalagu N Bhola PD Chang W McBrayer SK. Developmental regulation of mitochondrial apoptosis by c-myc governs age- and tissue-specific sensitivity to cancer therapeutics. Cancer Cell. (2017) 31:142–56. 10.1016/j.ccell.2016.11.011 28017613 PMC5363285

[18] Wong YK Zhang JB Hua ZC Lin QS Shen HM Wang JG. Recent advances in quantitative and chemical proteomics for autophagy studies. Autophagy. (2017) 13:1472–86. 10.1080/15548627.2017.1313944 28820289 PMC5612287

[19] Su Z Yang Z Xu Y Chen Y Yu Q. Apoptosis, autophagy, necroptosis, and cancer metastasis. Mol Cancer. (2015) 14:48. 10.1186/s12943-015-0321-5 25743109 PMC4343053

[20] Polyakov N Leshina T Fedenok L Slepneva I Kirilyuk I Furso J. Redox-active quinone chelators: properties, mechanisms of action, cell delivery, and cell toxicity. Antioxid Redox Sign. (2018) 28:1394–403. 10.1089/ars.2017.7406 29161882

[21] Prasad S Gupta SC Tyagi AK. Reactive oxygen species (ROS) and cancer: role of antioxidative nutraceuticals. Cancer Lett. (2017) 387:95–105. 10.1016/j.canlet.2016.03.042 27037062

[22] Shen KK Xie JL Wang H Zhang H Yu MY Lu FF. Cambogin induces caspase-independent apoptosis through the ros/jnk pathway and epigenetic regulation in breast cancer cells. Mol Cancer Ther. (2015) 14:1738–49. 10.1158/1535-7163.Mct-14-1048 25976678

[23] Zhang Y Kwok-Shing Ng P Kucherlapati M Chen F Liu Y Tsang YH. A pan-cancer proteogenomic atlas of PI3K/AKT/mTOR pathway alterations. Cancer Cell. (2017) 31:820–32 e3. 10.1016/j.ccell.2017.04.013 28528867 PMC5502825

[24] Zhang N Zhang J Tan YQ Du GF Lu R Zhou G. Activated Akt/mTOR-autophagy in local T cells of oral lichen planus. Int Immunopharmacol. (2017) 48:84–90. 10.1016/j.intimp.2017.04.016 28482233

[25] Yu H Qiu Y Pang X Li J Wu S Yin S. Lycorine promotes autophagy and apoptosis via TCRP1/Akt/mTOR axis inactivation in human hepatocellular carcinoma. Mol Cancer Ther. (2017) 16:2711–23. 10.1158/1535-7163.MCT-17-0498 28974556

[26] McKinley KL Cheeseman IM. Large-Scale Analysis of CRISPR/Cas9 Cell-Cycle Knockouts reveals the diversity of p53-dependent responses to cell-cycle defects. Dev Cell. (2017) 40:405–20 e2. 10.1016/j.devcel.2017.01.012 28216383 PMC5345124

[27] Codogno P Morel E. FOXO3a provides a quickstep from autophagy inhibition to apoptosis in cancer therapy. Dev Cell. (2018) 44:537–9. 10.1016/j.devcel.2018.02.019 29533768

[28] Xu L Fan Q Wang X Zhao X Wang L. Inhibition of autophagy increased AGE/ROS-mediated apoptosis in mesangial cells. Cell Death Dis. (2016) 7:e2445. 10.1038/cddis.2016.322 27809300 PMC5260901

[29] Morel E Mehrpour M Botti J Dupont N Hamai A Nascimbeni AC. Autophagy: a druggable process. Annu Rev Pharmacol Toxicol. (2017) 57:375–98. 10.1146/annurev-pharmtox-010716-104936 28061686

[30] Ariosa AR Klionsky DJ. A novel role for a glycolytic pathway kinase in regulating autophagy has implications in cancer therapy. Autophagy. (2017) 13:1091–2. 10.1080/15548627.2017.1321723 28537472 PMC5529076

[31] Ichim G Tait SWG. A fate worse than death: apoptosis as an oncogenic process. Nat Rev Cancer. (2016) 16:539–48. 10.1038/nrc.2016.58 27364482

[32] Fitzwalter BE Towers CG Sullivan KD Andrysik Z Hoh M Ludwig M. Autophagy inhibition mediates apoptosis sensitization in cancer therapy by relieving FOXO3a turnover. Dev Cell. (2018) 44:555–65 e3. 10.1016/j.devcel.2018.02.014 29533771 PMC5866042

[33] Yoo BH Zagryazhskaya A Li Y Koomson A Khan IA Sasazuki T. Upregulation of ATG3 contributes to autophagy induced by the detachment of intestinal epithelial cells from the extracellular matrix, but promotes autophagy-independent apoptosis of the attached cells. Autophagy. (2015) 11:1230–46. 10.1080/15548627.2015.1056968 26061804 PMC4590629

[34] Mills CC Kolb EA Sampson VB. Recent advances of cell-cycle inhibitor therapies for pediatric cancer. Cancer Res. (2017) 77:6489–98. 10.1158/0008-5472.Can-17-2066 29097609 PMC5712276

[35] Hu X Moscinski LC Cdc2: a monopotent or pluripotent CDK? Cell Prolif. (2011) 44:205–11. 10.1111/j.1365-2184.2011.00753.x 21535261 PMC6496858

[36] Boutros R Lobjois V Ducommun B. CDC25 phosphatases in cancer cells: key players? Good targets? Nat Rev Cancer. (2007) 7:495–507. 10.1038/nrc2169 17568790

[37] Deng TG Yan GB Song X Xie L Zhou Y Li JL. Deubiquitylation and stabilization of p21 by USP11 is critical for cell-cycle progression and DNA damage responses. Proc Natl Acad Sci USA. (2018) 115:4678–83. 10.1073/pnas.1714938115 29666278 PMC5939064

[38] Yaacoub K Pedeux R Tarte K Guillaudeux T. Role of the tumor microenvironment in regulating apoptosis and cancer progression. Cancer Lett. (2016) 378:150–9. 10.1016/j.canlet.2016.05.012 27224890

[39] Pei J Deng J Ye Z Wang J Gou H Liu W. Absence of autophagy promotes apoptosis by modulating the ROS-dependent RLR signaling pathway in classical swine fever virus-infected cells. Autophagy. (2016) 12:1738–58. 10.1080/15548627.2016.1196318 27463126 PMC5079672

[40] Shi Y Han Y Xie F Wang A Feng X Li N. ASPP2 enhances oxaliplatin (L-OHP)-induced colorectal cancer cell apoptosis in a p53-independent manner by inhibiting cell autophagy. J Cell Mol Med. (2015) 19:535–43. 10.1111/jcmm.12435 25534115 PMC4369811

[41] Rah B ur Rasool R Nayak D Yousuf SK Mukherjee D Kumar LD. PAWR-mediated suppression of BCL2 promotes switching of 3-azido withaferin A (3-AWA)-induced autophagy to apoptosis in prostate cancer cells. Autophagy. (2015) 11:314–31. 10.1080/15548627.2015.1017182 25803782 PMC4502794

[42] Helfinger V Schroder K. Redox control in cancer development and progression. Mol Aspects Med. (2018) 63:88–98. 10.1016/j.mam.2018.02.003 29501614

[43] Zhang L Kim SB Luitel K Shay JW. Cholesterol depletion by TASIN-1 induces apoptotic cell death through the ER stress/ROS/JNK signaling in colon cancer cells. Mol Cancer Ther. (2018) 17:943–51. 10.1158/1535-7163.Mct-17-0887 29467273

