# Supplementary material for: Triptolide Induces Glioma Cell Autophagy and Apoptosis via Upregulating the ROS/JNK and Downregulating the Akt/mTOR Signaling Pathways
Source: Front Oncol. 2019 May 14;9:387. doi: 10.3389/fonc.2019.00387 (PMC6528693; doi:10.3389/fonc.2019.00387)
Supplement: Supplementary file 1 [file Data_Sheet_1.docx]

Supplementary Material

**Table S1. IC50 values of triptolide in glioma and astrocyte cells**

| Cell lines | U251 | U87-MG | C6 | Astrocyte |
| --- | --- | --- | --- | --- |
| IC50 (nM)-24h | 382.80 | 325.09 | 175.87 | 6356.90 |
| IC50 (nM)-48h | 71.94 | 72.58 | 54.48 | 432.70 |

IC50 values for each cell lines were calculated by using Graphpad software. One representative result from triplicate was shown.

**Fig. S1**


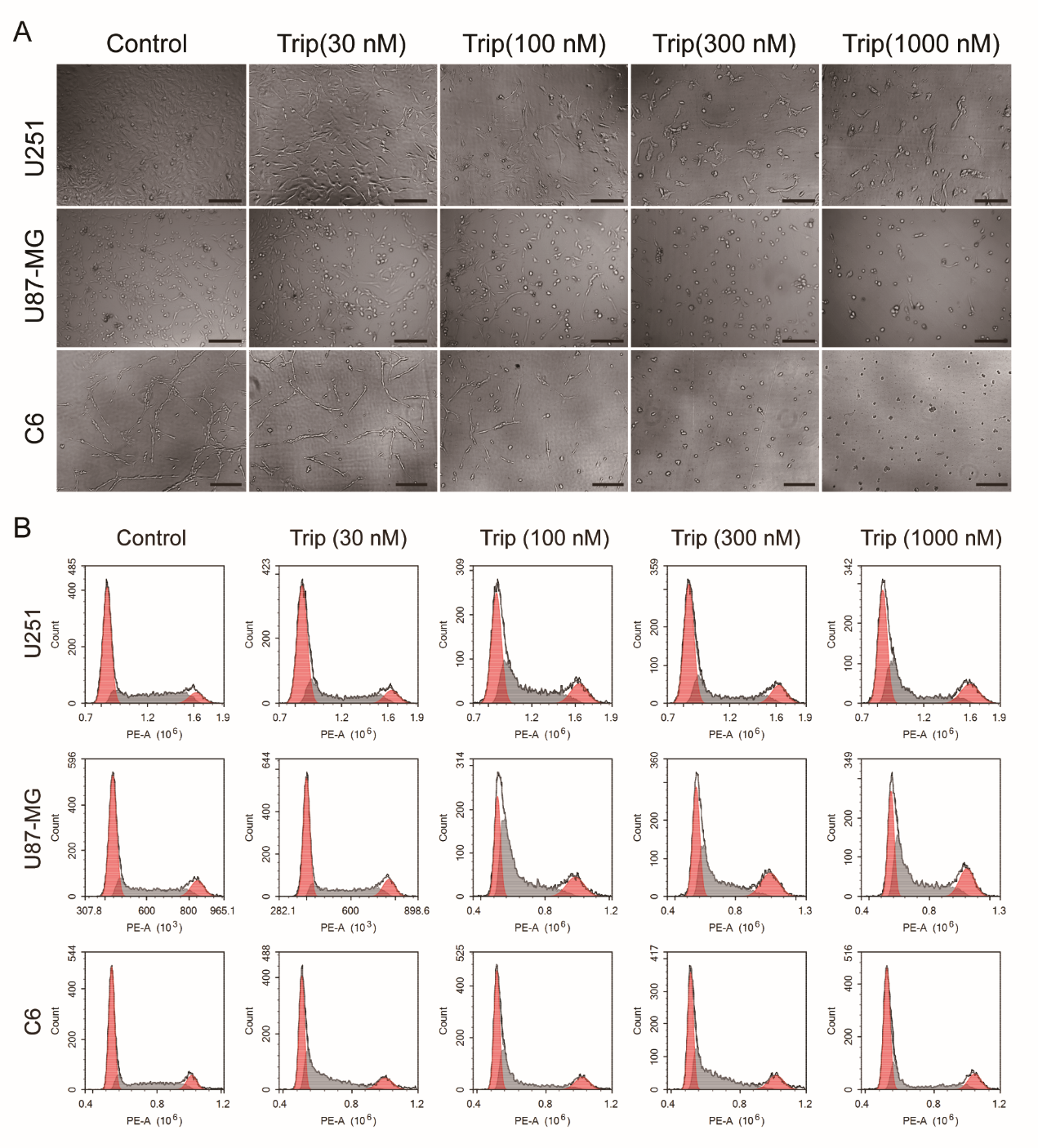


**Supplementary Figure 1.** Triptolide inhibited the proliferation of glioma cells and induced G2/M cell cycle arrest. (**A)** U251, U87-MG and C6 cells were treated with the indicated concentrations of triptolide or vehicle (DMSO) for 24 h. Representative images are shown as photographed with a phase contrast microscope (×200); scale bar:200 μm. **(B)** U251, U87-MG and C6 cells were treated with triptolide for 24 h and stained with PI. Cell cycle progression was analyzed by flow cytometry.

**Fig. S2**


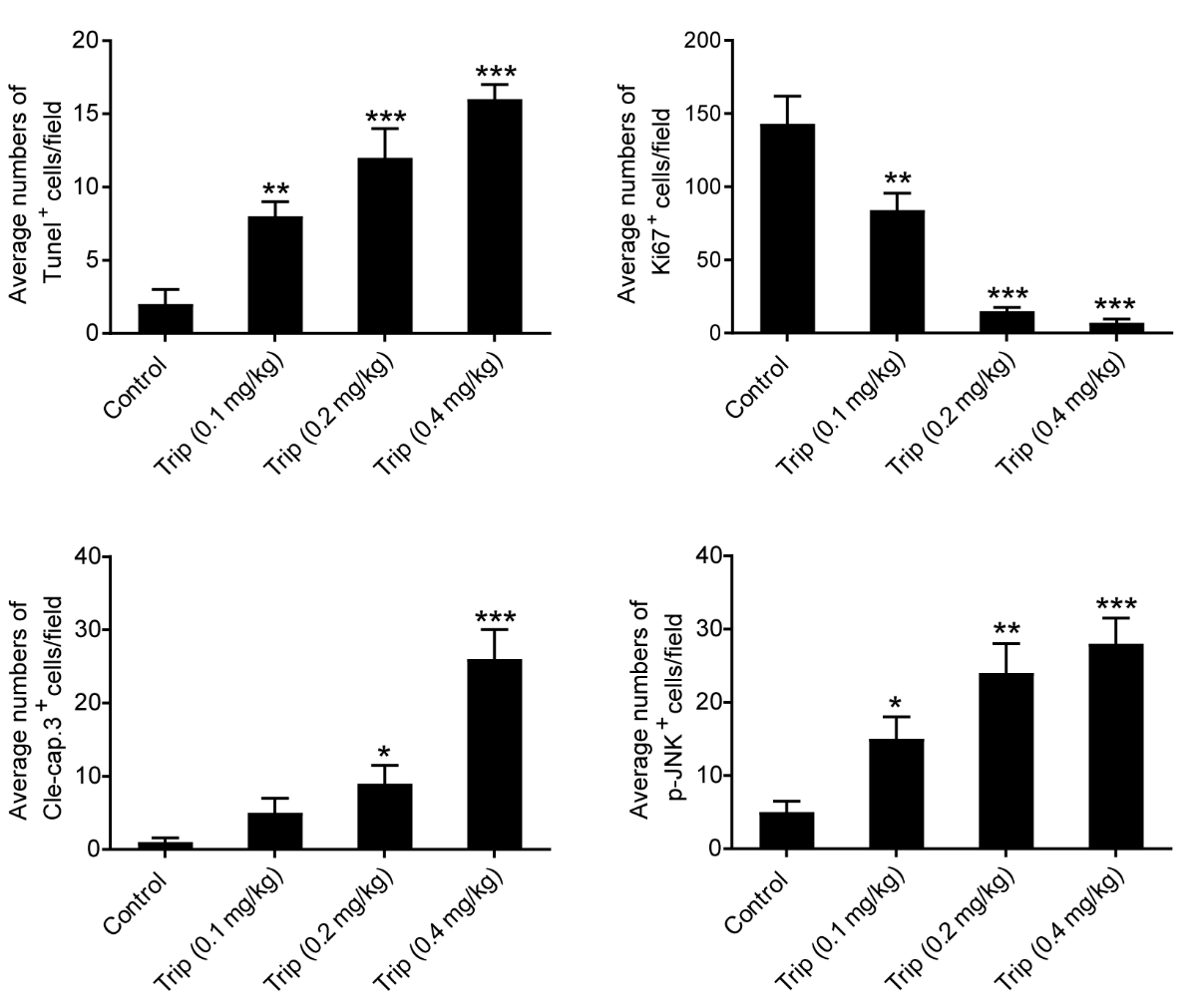


**Supplementary Figure 2.** The quantitative results of TUNEL and immunohistochemical staining of tumor specimens. The data are presented as the means ± SD (n=3). **P*<0.05, ***P*<0.01, ****P*<0.001, significantly different compared with the untreated control group.
